# A scalable, aggregated genotypic–phenotypic database for human disease variation

**DOI:** 10.1093/database/baz013

**Published:** 2019-02-13

**Authors:** Ryan Barrett, Cynthia L Neben, Anjali D Zimmer, Gilad Mishne, Wendy McKennon, Alicia Y Zhou, Jeremy Ginsberg

**Affiliations:** Color Genomics, 831 Mitten Road, Suite 100, Burlingame, CA, USA

## Abstract

Next generation sequencing multi-gene panels have greatly improved the diagnostic yield and cost effectiveness of genetic testing and are rapidly being integrated into the clinic for hereditary cancer risk. With this technology comes a dramatic increase in the volume, type and complexity of data. This invaluable data though is too often buried or inaccessible to researchers, especially to those without strong analytical or programming skills. To effectively share comprehensive, integrated genotypic–phenotypic data, we built Color Data, a publicly available, cloud-based database that supports broad access and data literacy. The database is composed of 50 000 individuals who were sequenced for 30 genes associated with hereditary cancer risk and provides useful information on allele frequency and variant classification, as well as associated phenotypic information such as demographics and personal and family history. Our user-friendly interface allows researchers to easily execute their own queries with filtering, and the results of queries can be shared and/or downloaded. The rapid and broad dissemination of these research results will help increase the value of, and reduce the waste in, scientific resources and data. Furthermore, the database is able to quickly scale and support integration of additional genes and human hereditary conditions. We hope that this database will help researchers and scientists explore genotype–phenotype correlations in hereditary cancer, identify novel variants for functional analysis and enable data-driven drug discovery and development.

## Introduction

Next generation sequencing (NGS) technologies continue to revolutionize the field of genomics as low-cost, high-throughput platforms with high sensitivity. Over the past few years, NGS multi-gene panels have been increasingly used in both the clinic and research laboratories for genetic screening, diagnosis and assessment of hereditary conditions, including cancer (1–3). About 10–15% of common cancers have been associated with inherited pathogenic or likely pathogenic variants that have well-established clinical presentations (4); an additional 5–15% are thought to be inherited (i.e. familial), but the underlying genetic etiologies have yet to be identified (5, 6). The study of genomic data in these cases can help reveal genotype–phenotype correlations in hereditary cancer, identify novel variants for functional analysis and enable data-driven drug discovery and development. However, the expanding volume, type and complexity of such data pose several bioinformatics challenges in storage, analysis and interpretation (7).

**Figure 1:**
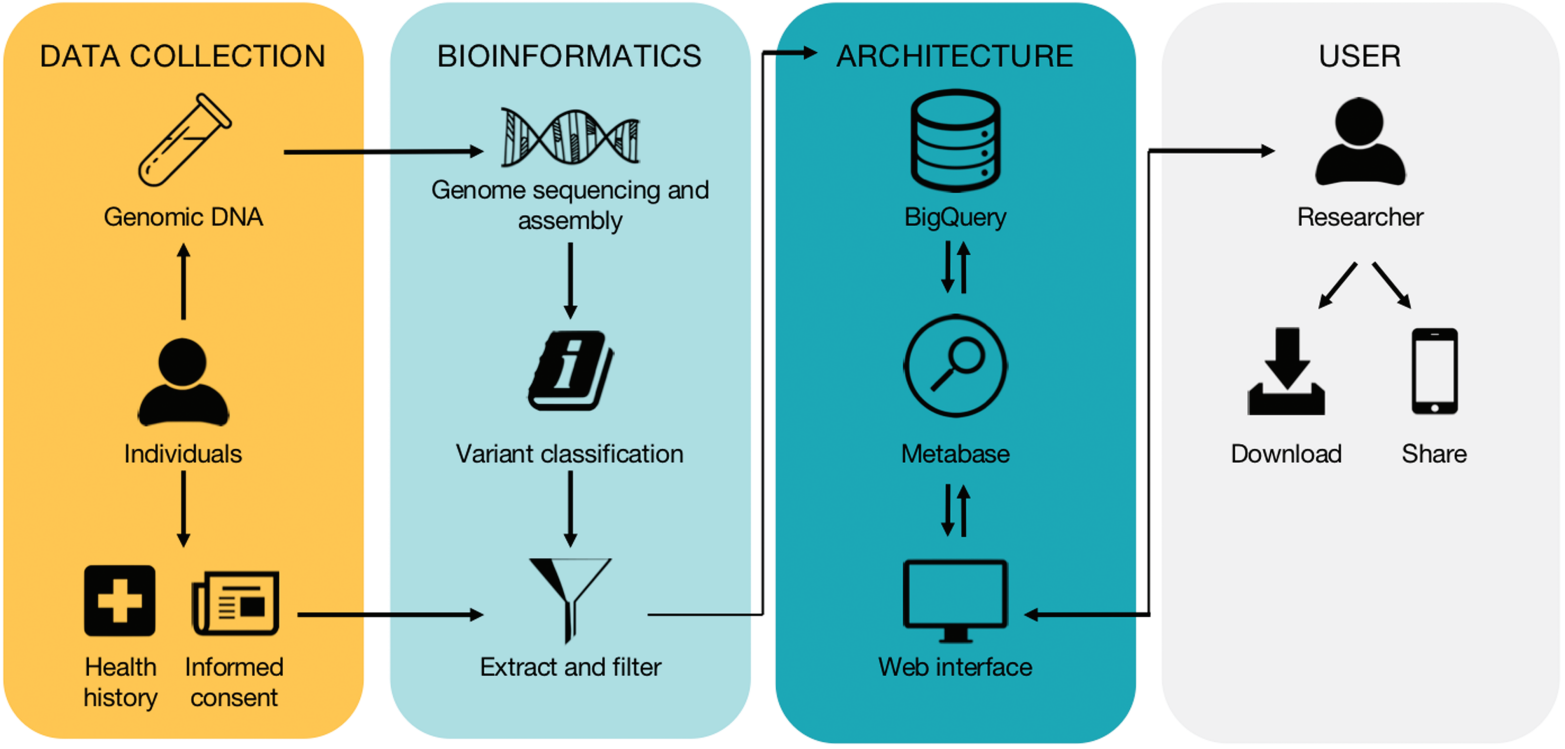
High-level workflow of the database. The workflow is divided into four subwork processes including ‘Data Collection’, ‘Bioinformatics’, ‘Architecture’ and ‘User’, grouped by four different color-rounded rectangles.

Several public population databases, as well as public and commercial cancer-specific databases, have been developed for genomic data and provide useful information on gene annotation, allele frequency and known or predicted functional consequences of variants (8–11). The sharing and pooling of this data is critical in interpreting the clinical significance of variants and delivery of genomic medicine (11, 12). However, associated specific clinical information, such as demographics and personal and family history, is not always available, and independently linking large sets of genotypic and phenotypic information often require knowledge of programming languages and database intelligence or expensive local software.

To effectively share comprehensive, integrated genotypic–phenotypic data, we built Color Data, a cloud-based database that supports broad access and data literacy. Our user-friendly interface allows researchers to easily execute their own queries with filtering. The results of queries are visualized as text and graphic features and can be downloaded in tabular format directly through the database to conduct further data analysis. At the time of publication, the database contains gene variants and phenotypes from 50 000 affected and unaffected individuals who were sequenced for 30 genes associated with hereditary cancer risk. Importantly, we have designed the web interface and underlying implementation to quickly scale and support samples and information from millions of individuals, as well as whole-genome sequencing data.

## Materials and methods

The high-level workflow and technical overview of the database are depicted in Figure 1 and described in detail below.

### Data collection

Individuals were ordered a Color test by a healthcare provider. All phenotypic information was reported by the individual through an interactive, online health history tool in their Color account. Phenotypic questions asked are available upon request. Individuals who reported more than one ancestry were counted as ‘Multiple ethnicities’ with the following exceptions: (i) any individuals who reported ‘Ashkenazi Jewish’ in addition to any other ancestry were counted as ‘Ashkenazi Jewish’; (ii) any individuals who reported ‘Hawaiian’ were counted as ‘Pacific Islander’; and (iii) any individuals who reported any combination of ‘Chinese’, ‘Japanese’, ‘Indian’, ‘Filipino’, ‘Hawaiian’, ‘Other Pacific Islander’ or ‘Other Asian’ and no other ancestry were counted as ‘Asian, not specified’.

All individuals consented to have their information appear in Color’s research database. Individuals were not recruited for this database and can opt out of participating in the database. This population was not specifically selected for any particular metric including gender, age, ethnicity or history of cancer, and individuals were included in consecutive order.

### Bioinformatics pipeline

Laboratory procedures were performed at the Color laboratory (Burlingame, CA) under Clinical Laboratory Improvements Amendments (#05D2081492) and College of American Pathologists (#8975161) compliance as previously described (3). Briefly, genomic DNA was extracted from blood or saliva (Perkin Elmer Chemagic DNA Extraction Kit), enriched for select regions using SureSelect XT probes and then sequenced using NextSeq 500/550 or NovaSeq 6000 instruments (Illumina). Sequence reads were aligned against human genome reference GRCh37.p12 with the Burrows–Wheeler Aligner (13), and duplicate and low quality reads were removed. Single nucleotide variants and small insertions and deletions (indels, 2–50 bp) were called by the HaplotypeCaller module of GATK3.4 (14). Variants in homopolymer regions were called by an internally developed algorithm using SAMtools. Large structural variants (>50 bp) were detected using dedicated algorithms based on read depth (CNVkit) (15), paired reads and split reads [LUMPY (16), in-house developed algorithms].

Variants were classified according to the standards and guidelines for sequence variant interpretation of the American College of Medical Genetics and Genomics (17), and all variant classifications were signed out by board certified medical geneticist or pathologist. Variant classification categories are pathogenic (P), likely pathogenic (LP), variant of uncertain significance (VUS), likely benign (LB) and benign (B).

The genes in Color Data were selected based on (i) published evidence of their association with hereditary cancer risk and (ii) the technical feasibility of sequencing them using the NGS methods described above. These genes are *APC, ATM, BAP1, BARD1, BMPR1A, BRCA1, BRCA2, BRIP1, CDH1, CDK4, CDKN2A* (p14ARF and p16INK4a), *CHEK2, EPCAM, GREM1, MITF, MLH1, MSH2, MSH6, MUTYH, NBN, PALB2, PMS2, POLD1, POLE, PTEN, RAD51C, RAD51D, SMAD4, STK11* and *TP53*. Analysis, variant calling and reporting focused on the complete coding sequence and adjacent intronic sequence of the primary transcript(s) (Table S1), unless otherwise indicated. In *PMS2*, exons 12–15 were not analyzed. In several genes, only specific positions known to impact cancer risk were analyzed (genomic coordinates in GRCh37): *CDK4*—only chr12:g.58145429-58145431 (codon 24), *MITF—*only chr3:g.70014091 (including c.952G>A), *POLD1—*only chr19:g.50909713 (including c.1433G>A), *POLE—*only chr12:g.133250250 (including c.1270C>G), *EPCAM*—only large deletions and duplications including the 3′ end of the gene and *GREM1—*only duplications in the upstream regulatory region.

### Architecture and implementation

Color Data is static HTML and CSS with Metabase embedded in <iframe>s in the web interface to allow users to perform data analysis. The HTML/CSS is served by Amazon Web Services S3 and CloudFront, which provide a secure cloud services platform, computing power and scalability. The data is stored in Google BigQuery, another widely used cloud-based data warehouse, in a dedicated project. The underlying tables are generated by extract, transform and load (ETL); this ETL process also performs filtering for inclusion and exclusion from the database (Table **1**). At the time of publication, filtering for inclusion and exclusion from the database eliminated 2.5% of the source data.

**Table 1:** Criteria for inclusion and exclusion

Input	Inclusion	Exclusion
Individual	Referred by health care provider order for a Color test	Participant in another research study
	Informed consent	>10 missing phenotype data points^a^
	Sample passed internal quality control	
Phenotype data	Reported health history via online Color account	Reported event age > current individual age
	Reported age, gender, number of children, number of siblings (unless reported to be adopted)	For numeric data points: An absolute modified Z-score > 5 or above Q3 + 3^*^IQR or below Q1 − 3^*^IQR
Genotype data	Sequenced for 30 genes associated with hereditary cancer risk	For variants in *SMAD4*:^b^ Allele frequency < 0.3
	Read depth ≥ 20If variant sent for secondary confirmation, confirmed presentVariant classification submitted to ClinVar	For variants in genes other than *SMAD4*: Allele frequency < 0.2 and reference allele or alternate allele length < 10 bpCalled only by Scalpel

^a^If an individual has >10 phenotype data points missing, that individual is excluded from the database.

^b^ *SMAD4* has a common processed pseudogene, which may result in artifacts at lower allele fractions. Q, quartile. IQR, interquartile range.

The database is powered by Metabase, an open source data analysis tool developed by Metabase Inc. and licensed under the AGPL v3. It runs on a dedicated site and accesses Google BigQuery via its REST API over HTTPS. Importantly, Metabase’s easy-to-use graphical user interface (GUI) allows users to run queries and visualize results without technical knowledge of computer programming or Structured Query Language (SQL) database query language.

The database URL includes a version (v) identifier that is assigned in increasing order and corresponds to new developments in the database. A new version will be assigned when there are significant changes to the data (in quantity or composition), inputs and outputs, filters and other functionalities. Users who cite the database should include the version identifier from which they derived their results as queries may change between versions. Importantly, the data and functionality within a version will remain fixed so that queries may be reproduced and replicated regardless of the current version.

### Privacy

To help protect the privacy of individuals whose information is included in Color Data, all information in the database is de-identified in compliance with the HIPAA Privacy Rule and is returned in aggregate. We took additional steps to limit re-identification of a single individual while still maintaining the power of aggregate and statistical database queries. These precautions were largely inspired by the literature on statistical databases (18, 19), differential privacy (20, 21) and Hippocratic databases (22). Query filters such as age are quantized into five-year buckets, and all queries are required to match at least 5 individuals, or results will not be returned, and an error message will be generated. Taken together, these restrictions can help to stymie some common techniques used to re-identify individuals in de-identified, aggregate data sets:

1. Using known incorrect or outlier data to match and identify a single target individual;
2. Using overly narrow query filters to match and identify a single target individual;
3. Finding two queries (A and B) where B matches everyone in A and a target individual and then subtracting the results of A from B to match and identify the target;
4. Finding two queries (C and D) where the only individual in common is a target and then intersecting their results to identify that target; and
5. Using multiple mean and median queries that overlap with known sets of individual(s) to calculate the differences to re-identify the overlapping individual(s).

Finally, all queries in the database and their source IP addresses are logged to detect, and potentially block, users who are making many suspiciously overlapping queries.

## Results

### Web interface

The home page (https://data.color.com/) introduces users to Color Data with a summary statement about the database, a ‘Get started’ button to the query/results page, sample queries of the data set (discussed here below) and links to ‘About Color Data’ and ‘FAQs’ pages. The ‘About Color Data’ page (https://data.color.com/about.html) contains up-to-date details about the scientific methodology and design of the database, which at the time of publication correspond with the Materials and Methods described here. The ‘FAQs’ page (https://data.color.com/faq.html) was designed to be accessible to a broad audience interested in human hereditary conditions including researchers and scientists, health systems and individuals who consented to the use of their information in the database.

On the query/results page (https://data.color.com/v1/), users can apply query filters to focus the data to a more specific range of results. These filter categories and filter values are listed in Table **2**. To note, filter categories use ‘AND’ logic, and filter values within categories use ‘OR’ logic. Users can select filter values in the dropdown list or by text typing with autocomplete, with the exception of the ‘Variant’ filter values that can only be selected by text typing with autocomplete using Human Genome Variation Society (HGVS) nomenclature. Users can remove filter values by clicking the ‘x’ in the text field.

**Table 2:** Filter categories and filter values

Filter categories	Filter values
Gender	F, M
Age	18–25, 26–30, 31–35, 36–40, 41–45, 46–50, 51–55, 56–60, 61–65, 66–70, 71–75, 76–80, 81–85, 86–89, ≥90
Ethnicity	African, Ashkenazi Jewish, Asian, not specified; Caucasian, Chinese, Filipino, Hispanic, Indian, Japanese, Multiple ethnicities, Native American, Pacific Islander, Unknown^a^
Personal cancer history	Breast, Colorectal, Gastric, Melanoma, No cancer, Ovarian, Pancreatic, Prostate, Uterine
Classification	Benign, Likely Benign, Likely Pathogenic, Pathogenic, VUS
Gene	*APC, ATM, BAP1, BARD1, BMPR1A, BRCA1, BRCA2, BRIP1, CDH1, CDK4, CDKN2A,* ^b^ *CHEK2, EPCAM, GREM1, MITF, MLH1, MSH2, MSH6, MUTYH, NBN, PALB2, PMS2, POLD1, POLE, PTEN, RAD51C, RAD51D, SMAD4, STK11*, *TP53*
Variant	(Search by Nomenclature)^c^
Zygosity	Heterozygous, Homozygous

^a^Unknown includes information not reported.

^b^The *CDKN2A* locus encodes two gene products, p14ARF and p16INK4a.

^c^Filter values for ‘Variant’ can only be selected by text typing with autocomplete using HGVS nomenclature.

F, female. M, male. VUS, variant of uncertain significance.

Results in the web interface are visualized as text and graphic features to provide quick interpretation of information. Graphic features use a linear or power y-axis to achieve maximal data visualization. The ‘Percentage of Color Data Population’ is displayed for all queries. Query results can be broadly categorized into two types of data: genotypic and phenotypic. Genotypic data returned through the database include the ‘Pathogenic Frequency’, ‘Unique Variants by classification’, ‘Total Variants’, ‘Variants’, ‘Most Commonly Co-occurring P/LP Variants’ and ‘Most Commonly Co-occurring VUS/LB/B Variants’. To note, for ‘Most Commonly Co-occurring P/LP Variants’ and ‘Most Commonly Co-occurring VUS/LB/B Variants’, results will only be displayed when a user applies a single ‘Variant’ filter value. Phenotypic data returned through the database include ‘Gender’, ‘Mean age (Years)’, ‘Age’, ‘Ethnicity’, ‘Personal History of Cancer’, ‘Mean Onset Age’, ‘Individuals with Onset < 50 years’, ‘Cancer Onset Age Spectrum’ and ‘Family History of Cancer’. Any query where the return of results would yield information about <5 individuals will generate the following error message: ‘Too few individuals in the Color Data population match this query to return results.’

Users can download full results in csv, xlsx and json format directly from the query/results page to permanently store on their computer in tabular format. In addition, users can share queries and results via email or social media, including Facebook and Twitter, through integrated share buttons to facilitate the rapid and broad dissemination of information (Figure 1).

### Population characteristics

Approximately three-quarters of individuals in the database were women (79.61%), over age 40 years (67.66%) and Caucasian (72.12%). A total of 39 890 (79.78%) individuals reported no personal history of eight common hereditary cancers, while 3925 (7.85%) individuals had a personal history of breast cancer, 1351 (2.70%) had prostate cancer, 937 (1.87%) had melanoma and 1074 (2.15%) individuals had a personal history of another common hereditary cancer. The youngest mean onset age was melanoma at 46.13 ± 14.48 years compared to the highest mean onset age for pancreatic cancer at age 63.54 ± 10.78 years. The most commonly reported family history of cancer was breast, including 9769 mothers, 142 fathers, 9558 grandparents, 41 brothers, 3363 sisters and 388 children, followed by colorectal and prostate cancer.

Among 50 000 individuals, a total of 2 868 593 variants were identified in 25 genes, with the largest percentages in *BRCA2, BRCA1* and *APC.* No variants were identified in *EPCAM, CDK4, GREM1, POLD1* and *POLE*. The most common variant was the benign homozygous variant *ATM* c.5948A>G in 50 000 individuals. There were 11 348 unique variants, nearly half of which were benign or likely benign (4880). The frequency of pathogenic variants in the total population was 10.80%.

### Sample query 1: women with a personal history of breast cancer

**Figure 2:**
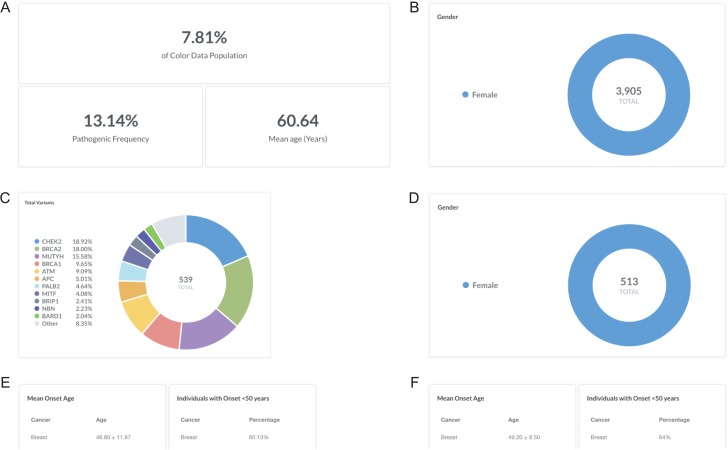
Screenshots of query results for the pathogenic frequency and cancer age of onset in women with breast cancer. (**A**, **B**) Filter by ‘Gender: F’ and ‘Cancer history: Breast’. (**C**, **D**) Filter by ‘Classification: Pathogenic or Likely Pathogenic’. (**E**) Filter by ‘Gene: *BRCA1* or *BRCA2*’. (**F**) Remove ‘Gene: *BRCA1* or *BRCA2*’ and filter by ‘Gene: *PALB2*’. Query URL: https://data.color.com/v1/#gender=F&cancer_history=Breast

Breast cancer is the most common cancer in women worldwide, with an average lifetime risk of 12% for women in the United States (18). While most hereditary cases of breast cancer are associated with pathogenic variants in *BRCA1* and *BRCA2*, less common variants in other genes have also been associated with increased risk of developing breast cancer (19, 20). To investigate the pathogenic frequency in females with breast cancer in the database, users can filter by ‘Gender: F’ and ‘Cancer history: Breast’ (https://data.color.com/v1/#gender=F&cancer_history=Breast); females with a personal history of cancer accounted for 7.81% of the database population with a pathogenic frequency of 13.14% (Figure 2A and B). To investigate the spectrum of pathogenic variants, users can filter by ‘Classification: Pathogenic or Likely Pathogenic’; a total of 539 pathogenic or likely pathogenic variants were identified in 513 women, nearly one-third of which were in *BRCA1* and *BRCA2* (Figure 2C and D)*.* To note, 252 of the total pathogenic or likely pathogenic variants were unique. To investigate the phenotypic information associated with each gene, users can filter by ‘Gene’. For example, 60.13% of the ‘Gene: *BRCA1* or *BRCA2*’ subpopulation had breast cancer before age 50 years, with a mean onset age at 46.80 ± 11.87 years (Figure 2E) compared to 64% of the ‘Gene: *PALB2*’ subpopulation that had breast cancer before age 50 years, with a mean onset age at 49.20 ± 8.50 years (Figure 2F). Taken together, users could use the result of this query as support for previous reports that pathogenic variants in *PALB2* are associated with breast cancer and that the risk may overlap with that for pathogenic variants in *BRCA2* (21–23).

### Sample query 2: Ashkenazi Jewish *BRCA1* and *BRCA2* founder alleles

**Figure 3:**
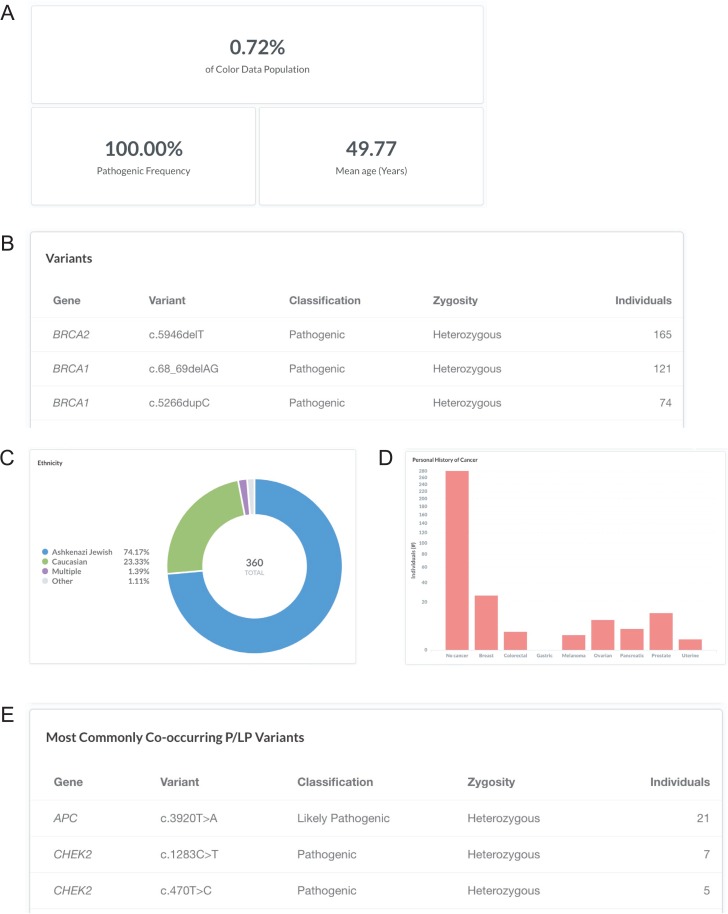
Screenshots of query results for the Ashkenazi Jewish BRCA founder alleles. (**A–E**) Filter by ‘Variant: c.68_69delAG, c.5266dupC, or c.5946delT’. Ashkenazi Jewish: the BRCA founder alleles are *BRCA1* c.68_69delAG, *BRCA1* c.5266dupC and *BRCA2* c.5946delT. Query URL: https://data.color.com/v1/#variant=c.68_69delAG&variant=c.5266dupC&variant=c.5946delT

Breast cancer risk is slightly higher among women of Ashkenazi Jewish descent than among other women, likely due to the high prevalence of *BRCA1* and *BRCA2* pathogenic or likely pathogenic variants in this population (24). Specifically, three founder alleles in *BRCA1* and *BRCA2* are collectively present in about 2.5% of Ashkenazi Jewish individuals but rarely occur in other ethnic populations: *BRCA1* c.68_69delAG (p.Glu23Valfs), *BRCA1* c.5266dupC (p.Gln1756Profs) and *BRCA2* c.5946delT (p.Ser1982Argfs) (25–27). To investigate the frequency of the Ashkenazi Jewish BRCA founder alleles in the database, users can filter by ‘Variant: c.68_69delAG, c.5266dupC, or c.5946delT’ (https://data.color.com/v1/#variant=c.68_69delAG&variant=c.5266dupC&variant=c.5946delT); the Ashkenazi Jewish BRCA founder alleles were identified in 360 individuals in the database population (*BRCA2* c.5946delT, 165; *BRCA1* c.68_69delAG, 121; *BRCA1* c.5266dupC, 74) with a mean age of 49.77 years (Figure 3A and B). Of these individuals, 74.17% were of Ashkenazi Jewish descent, whereas 23.33% of individuals were Caucasian, and 2.50% of individuals were Asian, multiple ethnicities or unknown (Figure 3C). More than three-quarters of individuals reported no personal history of eight common hereditary cancers (Figure 3D). The most commonly co-occurring pathogenic or likely pathogenic variants were *APC* c.3920T>A (p.Ile1307Lys), *CHEK2* c.1283C>T (p.Ser428Phe) and *CHEK2* c.470T>C (p.Ile157Thr) (Figure 3E), which are other known Ashkenazi Jewish founder alleles (28–30). Taken together, users could use the result of this query as support for previous reports that prioritizing genetic testing based on self-identity, such as for the Ashkenazi Jewish founder alleles, may miss pathogenic carriers (31, 32).

### Sample query 3: individuals with Lynch syndrome

**Figure 4:**
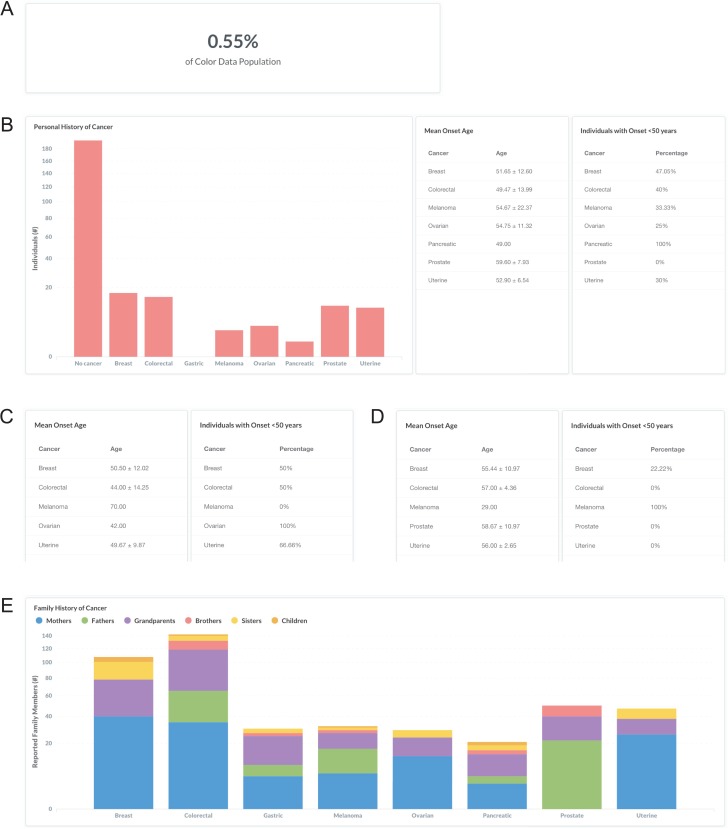
Screenshots of query results the personal and family history of cancer in individuals with Lynch syndrome. (**A**, **B**) Filter by ‘Classification: Pathogenic or Likely Pathogenic’ and ‘Gene: *MLH1, MSH2, PMS2, MSH6,* or *EPCAM*’. (**C**) Remove ‘Gene: *MSH2, PMS2, MSH6,* or *EPCAM*’. (**D**) Remove ‘Gene: *MLH1*’ and filter by ‘Gene: *PMS2*’. (**E**) Filter by ‘Gene: *MLH1, MSH2, PMS2, MSH6,* or *EPCAM*’. Query URL: https://data.color.com/v1/#classification=Likely%20Pathogenic&classification=Pathogenic&gene=MSH6&gene=MLH1&gene=MSH2&gene=PMS2&gene=EPCAM

Lynch syndrome, also known as hereditary non-polyposis colorectal cancer, is characterized by an increased risk for colorectal cancer and endometrial cancer associated with pathogenic or likely pathogenic variants in DNA mismatch repair genes (*MLH1, MSH2, MSH6, PMS2)* and *EPCAM* (33). A clinical diagnosis of Lynch syndrome is suspected in individuals with a strong personal and family history (34). To investigate the personal and family history of cancer in individuals with Lynch syndrome in the database, users can filter by ‘Classification: Pathogenic or Likely Pathogenic’ and ‘Gene: *MLH1, MSH2, PMS2, MSH6,* or *EPCAM*’ (https://data.color.com/v1/#classification=Likely%20Pathogenic&classification=Pathogenic&gene=MSH6&gene=MLH1&gene=MSH2&gene=PMS2&gene=EPCAM); individuals with Lynch syndrome accounted for 0.55% of the database population (Figure 4A). Of these individuals, 15 reported a personal history of colorectal cancer, with a mean onset age at 49.47 ± 13.99 years, and 10 females reported a personal history of uterine (endometrial) cancer with a mean onset age at 52.90 ± 6.54 years (Figure 4B). To investigate if this phenotypic information varies by gene, users can filter by ‘Gene’. For example, 50% of the ‘Gene: *MLH1*’ subpopulation had colorectal cancer before age 50 years, with a mean onset age at 44 ± 14.25 years (Figure 4C), compared to 0% of the ‘Gene: *PMS2*’ subpopulation that had colorectal cancer before age 50 years, with a mean onset age at 57 ± 4.36 years (Figure 4D). The most commonly reported family history of cancer among individuals with any Lynch syndrome gene was colorectal, including 35 mothers, 30 fathers, 53 grandparents, 14 brothers, 8 sisters and 2 children, followed by breast and prostate cancer (Figure 4E). Taken together, users could use the results of this query as support for previous reports that cancer risks and mean age at diagnosis vary among the genes associated with Lynch syndrome (33).

## Discussion

The field of clinical cancer genetics is rapidly expanding, generating vast amounts of human genomic data to be stored, analyzed and interpreted. In this paper, we described the development and use of Color Data, a cloud-based database that contains genotypic and phenotypic information from 50 000 individuals who were sequenced for 30 genes associated with hereditary cancer. Compared to other public population databases that provide robustly annotated sequencing data and allele frequencies and effects (8–11), this database focuses on genotypic–phenotypic correlations in hereditary cancer in an aggregated population and includes information of genes, variants and classifications, as well as personal and family history of cancer. It builds off of existing public and commercial cancer-specific databases but is distinctly novel in both the size and diversity of the population, as well as its ease of use for querying with filters and visualizing results. The rapid and broad dissemination of these research results through sharing and downloading will help increase the value of, and reduce the waste in, scientific resources and data.

The database may be limited by selection bias for Caucasians and women. Indeed, previous studies have demonstrated an ascertainment bias for women in genetic testing for hereditary cancer (35–38). This could be due to the general bias of panels towards genes for hereditary breast and ovarian cancer or other social factors. The database is enriched for relatives, who were referred for genetic testing through a cascade screening program for hereditary cancer risk (39). It may also be limited by self-reporting of phenotypic information (in contrast to submitted by a healthcare provider), although previous studies have reported high accuracy of self-reported data (40, 41). Ethnicity, personal history of cancer and family history of cancer were not available for every individual and thus resulted in incomplete data sets.

In future versions of the database, we plan to provide additional genotypic and phenotypic data, including variant types and effects and filtering on family history of cancer. By utilizing cloud hosting and large-scale reuse techniques, the database is able to quickly scale and support integration of additional genes and human hereditary conditions, such as hereditary cardiovascular conditions and whole-genome sequencing data. Furthermore, we plan to allow researchers to create their own queries in raw SQL and/or in the Metabase GUI to ask broader and deeper questions of the underlying raw data.

### Data sharing statement

The data in this report are publicly available at Color Data (https://data.color.com/v1/). All reported variants have been submitted to ClinVar (https://www.ncbi.nlm.nih.gov/clinvar/submitters/505849/).

## Supplementary Material

Supplementary Data

## Notes

**Database URL:** https://data.color.com/
