# Supplementary material for: A scalable, aggregated genotypic–phenotypic database for human disease variation
Source: Database (Oxford). 2019 Feb 13;2019:baz013. doi: 10.1093/database/baz013 (PMC6372842; doi:10.1093/database/baz013)
Supplement: Supplementary Data [file barrett,_neben_et_al_table_s1_revised_baz013_supp.doc]

Table S1. Reportable transcripts

| **Gene** | **Ensembl gene ID** | **Ensembl transcript ID** |
| --- | --- | --- |
| *APC* | [ENSG00000134982](http://grch37.ensembl.org/homo_sapiens/Gene/Summary?db=core;g=ENSG00000134982) | [ENST00000257430.4](http://grch37.ensembl.org/homo_sapiens/Transcript/Summary?db=core;t=ENST00000257430.4) |
| *ATM* | [ENSG00000149311](http://grch37.ensembl.org/homo_sapiens/Gene/Summary?db=core;g=ENSG00000149311) | [ENST00000278616.4](http://grch37.ensembl.org/homo_sapiens/Transcript/Summary?db=core;t=ENST00000278616.4) |
| *BAP1* | [ENSG00000163930](http://grch37.ensembl.org/homo_sapiens/Gene/Summary?db=core;g=ENSG00000163930) | [ENST00000460680.1](http://grch37.ensembl.org/homo_sapiens/Transcript/Summary?db=core;t=ENST00000460680.1) |
| *BARD1* | [ENSG00000138376](http://grch37.ensembl.org/homo_sapiens/Gene/Summary?db=core;g=ENSG00000138376) | [ENST00000260947.4](http://grch37.ensembl.org/homo_sapiens/Transcript/Summary?db=core;t=ENST00000260947.4) |
| *BMPR1A* | [ENSG00000107779](http://grch37.ensembl.org/homo_sapiens/Gene/Summary?db=core;g=ENSG00000107779) | [ENST00000372037.3](http://grch37.ensembl.org/homo_sapiens/Transcript/Summary?db=core;t=ENST00000372037.3) |
| *BRCA1* | [ENSG00000012048](http://grch37.ensembl.org/homo_sapiens/Gene/Summary?db=core;g=ENSG00000012048) | [ENST00000357654.3](http://grch37.ensembl.org/homo_sapiens/Transcript/Summary?db=core;t=ENST00000357654.3) |
| *BRCA2* | [ENSG00000139618](http://grch37.ensembl.org/homo_sapiens/Gene/Summary?db=core;g=ENSG00000139618) | [ENST00000544455.1](http://grch37.ensembl.org/homo_sapiens/Transcript/Summary?db=core;t=ENST00000544455.1) |
| *BRIP1* | [ENSG00000136492](http://grch37.ensembl.org/homo_sapiens/Gene/Summary?db=core;g=ENSG00000136492) | [ENST00000259008.2](http://grch37.ensembl.org/homo_sapiens/Transcript/Summary?db=core;t=ENST00000259008.2) |
| *CDH1* | [ENSG00000039068](http://grch37.ensembl.org/homo_sapiens/Gene/Summary?db=core;g=ENSG00000039068) | [ENST00000261769.5](http://grch37.ensembl.org/homo_sapiens/Transcript/Summary?db=core;t=ENST00000261769.5) |
| *CDK4* | [ENSG00000135446](http://grch37.ensembl.org/homo_sapiens/Gene/Summary?db=core;g=ENSG00000135446) | [ENST00000257904.6](http://grch37.ensembl.org/homo_sapiens/Transcript/Summary?db=core;t=ENST00000257904.6) |
| *CDKN2A* | [ENSG00000147889](http://grch37.ensembl.org/homo_sapiens/Gene/Summary?db=core;g=ENSG00000147889) | (p16) [ENST00000304494.5](http://grch37.ensembl.org/homo_sapiens/Transcript/Summary?db=core;t=ENST00000304494.5), (p14) [ENST00000579755.3](http://grch37.ensembl.org/homo_sapiens/Transcript/Summary?db=core;t=ENST00000579755.3) |
| *CHEK2* | [ENSG00000183765](http://grch37.ensembl.org/homo_sapiens/Gene/Summary?db=core;g=ENSG00000183765) | [ENST00000328354.6](http://grch37.ensembl.org/homo_sapiens/Transcript/Summary?db=core;t=ENST00000328354.6) |
| *EPCAM* | [ENSG00000119888](http://grch37.ensembl.org/homo_sapiens/Gene/Summary?db=core;g=ENSG00000119888) | [ENST00000263735.4](http://grch37.ensembl.org/homo_sapiens/Transcript/Summary?db=core;t=ENST00000263735.4) |
| *GREM1* | [ENSG00000166923](http://grch37.ensembl.org/Homo_sapiens/Gene/Summary?db=core;g=ENSG00000166923;r=15:33010175-33026870) | [ENST00000300177.4](http://grch37.ensembl.org/homo_sapiens/Transcript/Summary?db=core;t=ENST00000300177.4) |
| *MITF* | [ENSG00000187098](http://grch37.ensembl.org/homo_sapiens/Gene/Summary?db=core;g=ENSG00000187098) | [ENST00000394351.3](http://grch37.ensembl.org/homo_sapiens/Transcript/Summary?db=core;t=ENST00000394351.3) |
| *MLH1* | [ENSG00000076242](http://grch37.ensembl.org/homo_sapiens/Gene/Summary?db=core;g=ENSG00000076242) | [ENST00000231790.2](http://grch37.ensembl.org/homo_sapiens/Transcript/Summary?db=core;t=ENST00000231790.2) |
| *MSH2* | [ENSG00000095002](http://grch37.ensembl.org/homo_sapiens/Gene/Summary?db=core;g=ENSG00000095002) | [ENST00000233146.2](http://grch37.ensembl.org/homo_sapiens/Transcript/Summary?db=core;t=ENST00000233146.2) |
| *MSH6* | [ENSG00000116062](http://grch37.ensembl.org/homo_sapiens/Gene/Summary?db=core;g=ENSG00000116062) | [ENST00000234420.5](http://grch37.ensembl.org/homo_sapiens/Transcript/Summary?db=core;t=ENST00000234420.5) |
| *MUTYH* | [ENSG00000132781](http://grch37.ensembl.org/homo_sapiens/Gene/Summary?db=core;g=ENSG00000132781) | [ENST00000450313.1](http://grch37.ensembl.org/homo_sapiens/Transcript/Summary?db=core;t=ENST00000450313.1) |
| *NBN* | [ENSG00000104320](http://grch37.ensembl.org/homo_sapiens/Gene/Summary?db=core;g=ENSG00000104320) | [ENST00000265433.3](http://grch37.ensembl.org/homo_sapiens/Transcript/Summary?db=core;t=ENST00000265433.3) |
| *PALB2* | [ENSG00000083093](http://grch37.ensembl.org/homo_sapiens/Gene/Summary?db=core;g=ENSG00000083093) | [ENST00000261584.4](http://grch37.ensembl.org/homo_sapiens/Transcript/Summary?db=core;t=ENST00000261584.4) |
| *PMS2* | [ENSG00000122512](http://grch37.ensembl.org/homo_sapiens/Gene/Summary?db=core;g=ENSG00000122512) | [ENST00000265849.7](http://grch37.ensembl.org/homo_sapiens/Transcript/Summary?db=core;t=ENST00000265849.7) |
| *POLD1* | [ENSG00000062822](http://grch37.ensembl.org/homo_sapiens/Gene/Summary?db=core;g=ENSG00000062822) | [ENST00000440232.2](http://grch37.ensembl.org/homo_sapiens/Transcript/Summary?db=core;t=ENST00000440232.2) |
| *POLE* | [ENSG00000177084](http://grch37.ensembl.org/homo_sapiens/Gene/Summary?db=core;g=ENSG00000177084) | [ENST00000320574.5](http://grch37.ensembl.org/homo_sapiens/Transcript/Summary?db=core;t=ENST00000320574.5) |
| *PTEN* | [ENSG00000171862](http://grch37.ensembl.org/homo_sapiens/Gene/Summary?db=core;g=ENSG00000171862) | [ENST00000371953.3](http://grch37.ensembl.org/homo_sapiens/Transcript/Summary?db=core;t=ENST00000371953.3) |
| *RAD51C* | [ENSG00000108384](http://grch37.ensembl.org/homo_sapiens/Gene/Summary?db=core;g=ENSG00000108384) | [ENST00000337432.4](http://grch37.ensembl.org/homo_sapiens/Transcript/Summary?db=core;t=ENST00000337432.4) |
| *RAD51D* | [ENSG00000185379](http://grch37.ensembl.org/homo_sapiens/Gene/Summary?db=core;g=ENSG00000185379) | [ENST00000345365.6](http://grch37.ensembl.org/homo_sapiens/Transcript/Summary?db=core;t=ENST00000345365.6) |
| *SMAD4* | [ENSG00000141646](http://grch37.ensembl.org/homo_sapiens/Gene/Summary?db=core;g=ENSG00000141646) | [ENST00000342988.3](http://grch37.ensembl.org/homo_sapiens/Transcript/Summary?db=core;t=ENST00000342988.3) |
| *STK11* | [ENSG00000118046](http://grch37.ensembl.org/homo_sapiens/Gene/Summary?db=core;g=ENSG00000118046) | [ENST00000326873.7](http://grch37.ensembl.org/homo_sapiens/Transcript/Summary?db=core;t=ENST00000326873.7) |
| *TP53* | [ENSG00000141510](http://grch37.ensembl.org/homo_sapiens/Gene/Summary?db=core;g=ENSG00000141510) | [ENST00000269305.4](http://grch37.ensembl.org/homo_sapiens/Transcript/Summary?db=core;t=ENST00000269305.4) |
